# A Review on the Etiologies of the Development of Atrial Fibrillation After Cardiac Surgery

**DOI:** 10.3390/biom15030374

**Published:** 2025-03-05

**Authors:** Christos Ballas, Christos S. Katsouras, Christos Tourmousoglou, Konstantinos C. Siaravas, Ioannis Tzourtzos, Christos Alexiou

**Affiliations:** 1 Department of Cardiac Surgery, University Hospital of Ioannina, 45500 Ioannina, Greece; ballaschristos@gmail.com (C.B.); christostourmousoglou@gmail.com (C.T.); alexiou486@aol.com (C.A.); 2 1st Department of Cardiology, University Hospital of Ioannina, 45500 Ioannina, Greece; siaravaskon@gmail.com; 3 2nd Department of Cardiology, University Hospital of Ioannina, 45500 Ioannina, Greece; ioannistzourtz@gmail.com

**Keywords:** postoperative atrial fibrillation, cardiopulmonary bypass, aortic cross-clamping

## Abstract

Postoperative atrial fibrillation (POAF) is the most common arrhythmia following cardiac surgery. This review critically explores the interplay between cardiopulmonary bypass (CPB) and aortic cross-clamping (ACC) times in POAF development. CPB disrupts systemic homeostasis by inducing inflammatory cascades, oxidative stress, and ischemia–reperfusion injury. Prolonged ACC times further exacerbate myocardial ischemia and structural remodeling, with durations exceeding 60–75 min consistently linked to an increased POAF risk. However, variability in outcomes across studies reveals the complex, multifactorial nature of POAF pathogenesis. Patient-specific variables, such as baseline comorbidities and myocardial protection strategies, modulate these risks, emphasizing the need for personalized surgical approaches. Despite advancements in myocardial protection techniques and anti-inflammatory strategies, the incidence of POAF remains persistently high, indicating a gap in translating mechanistic insights into effective interventions. Emerging biomarkers, including microRNAs (e.g., miR-21, miR-483-5p, etc.) and markers of myocardial injury like troponin I, offer potential for enhanced risk stratification and targeted prevention. However, their clinical applicability requires further validation in diverse patient populations. This review underscores the critical need for integrative research that combines clinical, molecular, and procedural variables to elucidate the nuanced interplay of factors driving POAF. Future directions include leveraging advanced intraoperative monitoring tools, refining thresholds for CPB and ACC times, and developing individualized perioperative protocols.

## 1. Introduction

Postoperative atrial fibrillation (POAF) is the most common arrhythmia following cardiac surgery, affecting up to 65% of patients depending on the type of procedure and patient population. This arrhythmia is considered to arise from two principal mechanisms: enhanced automaticity in one or several rapidly depolarizing foci and re-entry involving one or more circuits within the atrial tissue. Combined coronary artery bypass grafting (CABG) and valvular procedures have the highest reported prevalence, affecting a spectrum between one-third and two-thirds of all patients, while rates for isolated CABG and valvular surgeries are slightly lower (30–40% of patients). Population characteristics that appear to increase its incidence are older age, previous history of atrial tachyarrhythmia, male gender, decreased left ventricular ejection fraction, left atrial enlargement, chronic obstructive pulmonary disease, chronic renal failure, diabetes mellitus, and rheumatic heart disease. POAF typically peaks within the first 2–3 postoperative days with around 70% of affected patients developing arrhythmia by the end of the fourth day and 94% by the sixth day. It is often transient, yet it carries significant clinical implications [1,2,3].

POAF is strongly associated with hemodynamic instability, stroke, and prolonged hospitalization, as well as increased rates of reoperation, infections, and organ dysfunction (such as respiratory failure and renal failure) [4,5]. In the long term, it independently predicts adverse outcomes, including a 2–4-fold higher risk of stroke and doubled all-cause mortality at 6 months [6]. These findings underscore the need to address its underlying mechanisms and perioperative risk factors.

Central to the development of POAF is cardiopulmonary bypass (CPB), a cornerstone of modern cardiac surgery that ensures oxygenation and circulation. However, CPB triggers systemic inflammation, oxidative stress, and ischemia–reperfusion injury, all of which promote atrial remodeling and arrhythmogenesis. Prolonged aortic cross-clamping (ACC) further compounds these risks, contributing to myocardial ischemia, low cardiac output syndrome, and renal dysfunction [7]. Despite its clear significance, the exact cross-clamping duration considered “safe” remains undefined, varying by patient and procedural factors, such as myocardial protection strategies and baseline cardiac function.

This review seeks to bridge critical gaps in the literature by synthesizing evidence on the impact of CPB and cross-clamping (CC) times on POAF. In doing so, it aims to provide insights into the mechanisms underlying this arrhythmia, highlight inconsistencies in reported outcomes, and propose future research directions to optimize surgical care and reduce POAF burden.

## 2. Methods

This review aims to systematically synthesize evidence on the impact of cardiopulmonary bypass and aortic cross-clamping durations on the development of postoperative atrial fibrillation. A comprehensive literature search was conducted using PubMed and Google Scholar, focusing on articles published between 2012 and 2024. The search incorporated keywords such as “Aortic Cross-Clamp Time”, “Cardiopulmonary Bypass”, “Pathophysiology of Postoperative Atrial Fibrillation”, “Aortic Cross-Clamp Time and Postoperative Atrial Fibrillation”, “Novel Markers for Postoperative Atrial Fibrillation”, and “Anti-inflammatory Interventions”.

To ensure the inclusion of the most relevant studies, the following criteria were utilized.

• Eligibility criteria were established, prioritizing the following:
  ○ Studies exploring the relationship between CPB or ACC durations and POAF.
  ○ Articles presenting data on inflammatory markers, myocardial injury, or arrhythmogenesis linked to ACC durations.
  ○ Randomized controlled trials and cohort studies published in English.
• Exclusion criteria included the following:
  ○ Case reports, conference abstracts, and articles without measurable outcomes related to POAF or ACC times.
  ○ Studies focusing exclusively on off-pump cardiac procedures without cross-clamping.

After screening and evaluating numerous articles, 12 studies were identified as highly relevant and included in this review. These studies were critically reviewed and their findings summarized in thematic tables. The selected studies directly addressed key aspects of POAF, including its association with CC time, systemic inflammation, and myocardial injury, providing a robust framework for thematic analysis.

## 3. Pathogenesis of POAF

POAF arises from a multifactorial combination of structural, electrophysiological, and systemic changes triggered by cardiac surgery. Its etiology reflects contributions from pre-existing patient factors, surgical techniques, and postoperative responses, culminating in complications such as thromboembolic events and hemodynamic instability. While often self-limiting, POAF significantly increases morbidity, prolongs hospital stays, and elevates the mortality risk [4,8,9].

### 3.1. Systemic Inflammation and Oxidative Stress

A central driver of POAF pathogenesis is systemic inflammation and oxidative stress, both exacerbated by CPB and surgical trauma. CPB induces a significant rise in inflammatory markers such as interleukin 6 (IL-6) and tumor necrosis factor alpha (TNF-α), with elevated neutrophil-to-lymphocyte ratio (NLR) and pan-immune inflammatory value (PIV) correlating with adverse outcomes, including prolonged intensive care unit (ICU) stays [10,11]. It is not clear whether they increase to a different extent compared to other types of surgery. Studies in lung transplantation further demonstrate CPB’s role in vascular injury through disrupted angiopoietin signaling and immune activation, although these findings are primarily pulmonary-focused [12].

Emerging strategies, such as Del Nido cardioplegia (DNC), may show promise in reducing myocardial injury during CPB. According to several recent studies, DNC appears to lower troponin and creatine kinase MB (CK-MB) levels, achieving a lower incidence of POAF compared to that when using another type of cardioplegia. However, there are also studies that contradict the reduced POAF incidence when using DNC, providing evidence of a more neutral relationship between these two factors [13,14,15,16]. These findings, alongside evidence linking on-pump CABG to a higher POAF incidence than off-pump procedures (29.09% vs. 10.74%), raise the suspicion of CPB playing a role in amplifying systemic inflammation and oxidative stress, particularly in high-risk patients with hypertension or vasopressor use [6,17,18].

Despite the strong association between inflammation and POAF, recent evidence suggests a nuanced relationship. For instance, a study observed no significant cytokine differences between patients with and without POAF, emphasizing the interplay of factors like age and CC time in arrhythmogenesis [19]. This complexity underscores the need for tailored approaches addressing inflammation alongside other perioperative variables.

### 3.2. Atrial Remodeling and Structural Changes

POAF development is further influenced by structural remodeling. Age-related changes, including myolysis, apoptosis, and fibrosis, create substrates for re-entry circuits, critical to arrhythmogenesis [20]. Biomarkers of fibrosis, such as pro-collagen I and pro-collagen III peptide, predict POAF risk, while therapies targeting profibrotic pathways, like microRNA-2 inhibition, hold promise [21,22].

### 3.3. Electrolyte Imbalances and Sympathetic Activation

Electrolyte imbalances also play a pivotal role. Hypomagnesemia and hypokalemia increase sinus node automaticity and cellular excitability, promoting arrhythmogenesis, though recent evidence questions their direct role in POAF risk [23,24]. An increased postoperative sympathetic activity amplifies calcium influx through L-type channels, further driving arrhythmogenic conditions. Beta-blockers targeting elevated postoperative heart rates (>80 bpm) have proven effective in mitigating these risks. The perioperative administration of amiodarone, which also has an effect on the sympathetic nervous system, reduces POAF risk, probably through similar mechanisms [25,26].

### 3.4. Integration and Future Directions

The pathogenesis of POAF reflects a complex network of interacting factors, including systemic inflammation, oxidative stress, structural remodeling, and sympathetic activation. While these mechanisms are well documented, inconsistencies across studies highlight the need for integrative research approaches. Tailored perioperative strategies that address patient-specific risks, surgical techniques, and postoperative care hold the potential to reduce POAF incidence. Future research should focus on the dynamic interplay between inflammation, myocardial stress, and atrial vulnerability, leveraging emerging biomarkers and advanced intraoperative monitoring tools.

### 3.5. Influence of CPB and ACC to Pathophysiological Changes

CPB ensures adequate oxygenation and circulation during cardiac surgery, but its complex management, including temperature regulation, fluid balance, and invasive monitoring, can have significant systemic effects. Evidence indicates that patients undergoing CABG with CPB face a higher risk of developing POAF compared to off-pump surgeries, emphasizing CPB’s potential role in POAF pathogenesis [27,28].

Central to these effects is the inflammatory response and oxidative stress triggered by CPB. Elevated levels of IL-6, TNF-α, and reactive oxygen species (ROS) are strongly associated with atrial remodeling and arrhythmogenesis. For instance, ROS production during CPB exacerbates oxidative damage to myocardial tissue, while endothelial dysfunction promotes inflammatory cascades, collectively increasing arrhythmia risk [17,18]. These processes are further compounded by disruptions in gap junction integrity and ion mishandling, which create conduction heterogeneity and predispose the atria to re-entry circuits [29].

During CPB and ACC, increased sympathetic activity and calcium mishandling in atrial myocytes amplify atrial extrasystoles. These premature electrical discharges, which often are observed before POAF episodes, exacerbate conduction heterogeneity and endo-epicardial asynchrony [30,31]. Prolonged CC durations further increase the risk of ischemia–reperfusion injury, systemic inflammation, and oxidative stress. A study on aortic valve replacement (AVR) identified CC times exceeding 150 min as independent predictors of morbidity, highlighting the systemic burden of prolonged ischemia [32].

The studies summarized in Table 1 emphasize the multifactorial impact of CPB and CC, with key findings linking systemic inflammatory responses, ROS production, and conduction abnormalities to POAF development.

Understanding the influence of CPB and CC on POAF underscores the need for targeted perioperative interventions. Optimizing bypass and ischemic times, along with integrating preventive strategies like antioxidant and anti-inflammatory therapies, can mitigate these deleterious effects. It has been already shown that the perioperative administration of antioxidants, such as N-acetylcysteine, polyunsaturated fatty acids, and drugs with anti-inflammatory properties such as colchicine, may have antiarrhythmic potential and reduce the incidence of POAF [33,34,35]. Therefore, future research should focus on refining surgical protocols and exploring novel biomarkers to better predict and prevent POAF in high-risk patients.

## 4. Influence of ACC Time on POAF

A prolonged ACC time emerges as a critical variable in the context of postoperative atrial fibrillation, as highlighted by its association with ischemic injury, systemic inflammation, and adverse outcomes. However, while several studies provide compelling evidence for the detrimental effects of extended ACC durations, others fail to establish a direct link, suggesting the need for a more nuanced interpretation of its role.

Evidence from studies correlating ACC durations exceeding 60–75 min with elevated risks of myocardial damage, systemic complications, and increased POAF incidence is robust. In detail, according to a multicenter study which included 2962 (2957 with a complete ACC time recording) patients who underwent isolated on-pump CABG, ACC durations longer than 75 min (n = 619), compared to durations ≤ 75 min (n = 2338), were associated with a significantly increased rate of in-hospital and 30-day mortality (n = 18 vs. n = 39, *p* = 0.002), the prolonged use of inotropes (n = 241 vs. n = 651, *p* < 0.0001), acute kidney injury (n = 179 vs. n = 506, *p* = 0.006), the postoperative use of short-term mechanical circulatory assist devices (n = 24 vs. n = 45, *p* = 0.024 for intra-aortic balloon pump and n = 6 vs. n = 4, *p* = 0.007 for extracorporeal membrane oxygenation device), and POAF (n = 174 vs. n = 595, *p* = 0.361, and n = 122/428 vs. n = 91/428, *p* = 0.023, after logistic regression analysis and propensity score matching, respectively). There was no mention of any perioperative use of b-blockers or other antiarrhythmic drugs, as well as the duration of the arrhythmia or its impact on the patients’ hemodynamic status. It was also not known whether the patients had a prior history of (paroxysmal) atrial fibrillation (AF) [36]. A small study of only 30 patients with isolated ischemic heart disease who underwent on-pump CABG showed that a low cardiac output postoperatively, as a sign of myocardial damage, prolonged mechanical ventilation time, and renal dysfunction, blood transfusions, prolonged hospital stay, and increased mortality are associated with an ACC time >60 min, independently. This study, although to a lesser extent due to the small sample of patients, may contribute to the theory of the association of increased ACC times with cardiac and systemic outcomes in these patients [37].

Elevated biomarkers such as troponin I further substantiate the physiological impact of prolonged ischemia, emphasizing the role of structural and electrophysiological remodeling in arrhythmogenesis. ACC durations above 50 min are associated with an increased mean troponin I value intraoperatively and on the first postoperative day in patients undergoing CABG operation, a phenomenon that is exacerbated when values >60 are noted, according to a 2019 study [38]. Additionally, long-term data revealing reduced survival rates associated with prolonged ACC underscore its significance beyond the immediate postoperative period. Based on the retrospective study of Swinkels et al., which included 456 consecutive patients who had undergone isolated aortic valve replacement, patients with an ACC time of less than 63 min had a non-statistically significant difference in the incidence of POAF compared to those with times ≥ 63 min (n = 53/226 vs. n = 73/230, *p* = 0.059) but showed reduced late survival (until 30 years after surgery), and indeed for every minute of increase in the ACC time, the hazard ratio for decreased late survival was 1.01 (95% CI = 1.00–1.02; *p*  =  0.012). Ιn this study, unlike the ACC time, the CPB time was not shown to be an independent predictor of decreased late survival [39].

Despite these findings, inconsistencies in other studies, particularly in low-risk populations, raise important questions about the interplay of ACC time with other perioperative factors. For instance, Dayi et al. studied the impact that various intraoperative factors (such as CPB duration, ACC time, hypothermia, the number of grafts, etc.) may have on the development of POAF in a group of 103 consecutive patients who underwent isolated CABG operation. They showed that none of them were statistically significantly associated with POAF. The same was proven for the following post-operative factors: ejection fraction, partial pressure of oxygen, hemoglobin, hematocrit, intubation time, ICU stay, value of creatinine, and values of various electrolytes [40]. A recent, single-center study, which included 100 patients with low-risk characteristics for developing atrial and ventricular tachyarrhythmias and bradyarrhythmias, revealed a weak correlation between the ACC duration and new onset of cardiac arrhythmia after cardiac surgery, implying a minimal relationship between these factors [41].

The heterogeneity in results may reflect the multifactorial nature of POAF, where the ACC time serves as one component of a broader network of contributing factors. For instance, while ischemia–reperfusion injury is a known driver of inflammation and atrial remodeling, patient-specific variables such as baseline comorbidities and surgical techniques likely modulate these effects. Moreover, the extreme cases of prolonged ACC, such as durations exceeding 500 min, demonstrate that advanced myocardial protection strategies can attenuate its impact, highlighting the importance of technical refinement and perioperative care [42].

To summarize the evidence linking CPB and ACC to the development of POAF, key studies are listed in Table 2. These studies highlight the multifactorial nature of POAF pathogenesis, with mechanisms ranging from ischemia–reperfusion injury to systemic inflammation and atrial remodeling.

These findings collectively suggest that, while the ACC duration is a significant factor in postoperative outcomes, its role in POAF cannot be fully isolated from the broader surgical and physiological contexts. Future research should aim to disentangle these complex interactions, integrating insights from diverse patient populations and advanced surgical practices. This approach may not only clarify the relative importance of the ACC time but also inform strategies to optimize perioperative care, balancing surgical precision with individualized patient management to mitigate the risks associated with prolonged ischemic durations.

### 4.1. Emerging Biomarkers and Predictors of POAF

POAF remains a frequent and multifactorial complication following cardiac surgery, influenced by systemic inflammation, myocardial injury, and atrial remodeling. Recent studies have identified key biomarkers and molecular pathways that contribute to its pathogenesis, shedding light on potential predictors and therapeutic targets.

### 4.2. Inflammatory Markers and POAF Risk

The NLR, a marker of systemic inflammation, has been studied in the context of cardiopulmonary bypass. Gosav et al. reported that an elevated baseline NLR serves as an independent predictor of “subacute POAF”, developing between the 5th and 30th postoperative days [43]. However, contrasting evidence from a meta-analysis by Liu et al., which included 12 studies and 9262 participants, found no significant association between the preoperative NLR and POAF after adjusting for covariates [44]. Similarly, Jacob et al. in a cohort of 277 patients undergoing elective cardiac surgery failed to establish a link between the preoperative NLR and secondary POAF. These discrepancies highlight the potential variability in inflammatory responses among patients and suggest that other markers, such as the neutrophil-to-white cell ratio (NWR), may be more predictive in specific populations, such as heart transplant recipients [45,46].

### 4.3. Myocardial Injury and Atrial Remodeling

The markers of myocardial injury, including troponin I, CK-MB, and high-sensitivity troponin T (hs-TnT), have emerged as critical predictors of POAF. Elevated postoperative hs-TnT levels were shown to correlate with the POAF risk in patients undergoing CABG surgery with CPB, while preoperative levels were not predictive. Interestingly, systemic C-reactive protein (CRP) levels, although elevated postoperatively, were not correlated with atrial tissue inflammation, suggesting a localized inflammatory process within the atria as the primary driver of arrhythmogenesis. This finding underscores the importance of ischemia-induced myocardial damage over systemic inflammatory markers in the development of POAF [47].

### 4.4. MicroRNAs and Molecular Insights

The role of microRNAs (miRNAs) in atrial remodeling and fibrosis has gained significant attention. miR-21 and miR-29a, in particular, have been implicated in regulating fibrosis and inflammation. miR-21 promotes atrial fibrosis through the signal cell adhesion molecule 1/transducer and activator of transcription 3 (CADM1/STAT3) pathway, making it a promising therapeutic target [48]. Conversely, miR-29a exhibits anti-fibrotic properties by downregulating Serpin Family H Member 1 (SERPINH1), a collagen chaperone, suggesting a protective role against cardiac fibrosis during aging [49]. Additionally, elevated miR-483-5p levels in preoperative serum demonstrated their superior diagnostic accuracy for POAF compared to traditional markers such as B-type natriuretic peptide (BNP) and CRP, offering a novel biomarker for early identification [50].

Other studies have explored the dynamic changes in miRNA levels during and after cardiac surgery. For instance, miR-1, miR-23a, and miR-26a were found to fluctuate postoperatively, with lower levels of miR-23a and miR-26a observed in POAF patients [51]. These findings suggest that miRNAs not only reflect the systemic and localized inflammatory milieu but also provide insights into the structural and molecular changes within the myocardium.

### 4.5. Proteomic and Metabolomic Predictors

Advancements in proteomics and metabolomics have identified additional pathways and biomarkers associated with POAF. The dysregulation of the peroxisome proliferator-activated receptor alpha (PPARα) pathway and glutathione metabolism was highlighted as the key contributor to POAF risk. Specific proteins, including apolipoprotein-C3 and glutathione peroxidase 3, were associated with redox imbalances, further emphasizing the interplay between oxidative stress and arrhythmogenesis [52].

The diverse findings from these studies underscore the multifactorial nature of POAF. While inflammatory markers like NLR and myocardial injury biomarkers such as hs-TnT provide valuable predictive insights, their inconsistent associations across studies highlight the need for individualized risk assessments. MiRNAs, particularly miR-21, miR-29a, and miR-483-5p, offer a promising avenue for early detection and therapeutic intervention, though their clinical application requires further validation. Proteomic and metabolomic approaches have added depth to our understanding, identifying novel pathways that may complement traditional risk factors.

**Figure 1:**
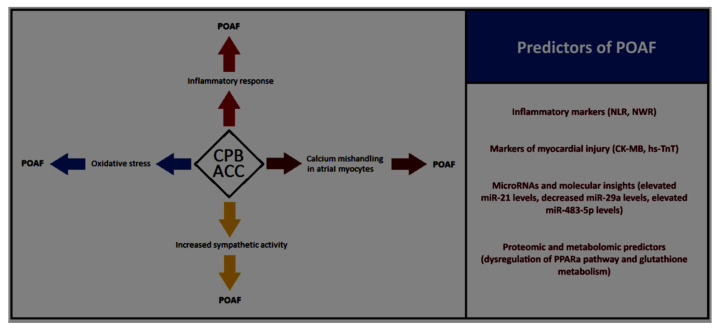
Influence of cardiopulmonary bypass (CPB) and aortic cross clamping (ACC) times on pathophysiological changes driving to postoperative atrial fibrillation (POAF) and the key clinical, biochemical, and molecular POAF’s predictors. NLR: neutrophil-to-lymphocyte ratio; NWR: neutrophil-to-white cell ratio; CK-MB: creatine kinase-MB; hs-TnT: high-sensitivity troponin T; PPARa: peroxisome proliferator-activated receptor alpha.

In conclusion, integrating clinical, biochemical, and molecular data holds the key to advancing POAF prediction (Figure 1) and management. Future research should focus on large-scale, multicenter trials to validate these findings and explore the synergistic use of biomarkers and genomics in risk stratification. By addressing these gaps, we can move closer to personalized approaches that mitigate the burden of POAF and improve patient outcomes.

## 5. Critical Analysis and Conclusions

POAF remains a persistent complication after cardiac surgery, with a multifactorial etiology that involves systemic inflammation, ischemia–reperfusion injury, oxidative stress, and myocardial remodeling. Despite decades of research and incremental advances, the impact of CPB and ACC on POAF underscores both the necessity and the risks of modern cardiac surgery.

The association between prolonged CPB and ACC times and the development of POAF reveals a complex interplay of physiological stressors. CPB, while indispensable for maintaining systemic circulation during surgery, triggers an inflammatory cascade marked by elevated cytokines such as IL-6 and TNF-α, along with oxidative stress, which contributes to atrial arrhythmogenesis [5,13]. Prolonged ACC times exacerbate ischemic injury, with durations exceeding 60–75 min linked to significant myocardial damage and adverse outcomes, such as an increased incidence of POAF and long-term complications like reduced cardiac function and survival [36,38].

These physiological disruptions are compounded by patient-specific factors, including pre-existing comorbidities like chronic obstructive pulmonary disease and hypertension, and procedural variables such as myocardial protection strategies. This variability highlights the need for personalized approaches to risk assessment and surgical planning.

While the current body of literature elucidates the mechanisms and risks associated with CPB and ACC, it often lacks granularity in addressing the heterogeneity of patient populations and procedural contexts. Emerging biomarkers, such as miRNAs (e.g., miR-21) and troponin I, show promise in enhancing risk stratification but are yet to see widespread clinical application [48,50]. Furthermore, despite advances in myocardial protection and anti-inflammatory strategies, the incidence of POAF has not seen a significant decline, emphasizing a gap in translating mechanistic insights into effective interventions.

To move the field forward, research should adopt a more integrative and dynamic approach. Large-scale, multicenter studies are needed to validate the predictive value of novel biomarkers and to refine thresholds for CPB and ACC times in diverse patient populations. Incorporating advanced monitoring tools that dynamically assess myocardial stress and inflammatory responses during surgery could enable real-time adjustments to mitigate risks. Additionally, personalized perioperative protocols, including tailored pharmacological interventions and optimized fluid management, hold potential to improve outcomes.

On a broader scale, understanding the interplay between systemic and localized atrial changes—such as fibrosis and electrical remodeling—remains a priority. Strategies that target these mechanisms, including antioxidant therapies and interventions that modulate the autonomic nervous system, warrant further exploration.

The role of CPB and ACC in POAF underscores the fine balance between the benefits and risks of cardiac surgery. While progress has been made in delineating the underlying mechanisms, achieving meaningful reductions in POAF incidence will require a shift toward personalized care and integrative research approaches. By bridging the gap between mechanistic insights and clinical practice, we can pave the way for more effective prevention and management strategies, ultimately improving surgical outcomes and patient quality of life.

## Figures and Tables

**Table 1:** The summary of studies examining the influence of cardiopulmonary bypass (CPB) and cross-clamping (CC) on pathophysiological changes relevant to postoperative atrial fibrillation (POAF). IL-6: interleukin 6; TNF-α: tumor necrosis factor alpha; ROS: reactive oxygen species; NLR: neutrophil-to-lymphocyte ratio; PIV: pan-immune inflammation value; ICU: intensive care unit.

Study	Focus	Key Findings
Jeong et al., 2012 [29]	Analyzed the disruption of gap junction integrity and ion mishandling in CPB	Gap junction disruptions and ion mishandling lead to conduction abnormalities
Qu et al., 2015 [18]	Focused on the endothelial dysfunction linked to oxidative stress in CPB	Endothelial dysfunction promotes inflammatory cascades, increasing arrhythmia risk
Zakkar et al., 2015 [17]	Investigated ROS production and its impact on myocardial tissue during CPB	ROS production leads to oxidative damage and exacerbates myocardial stress
Yadava et al., 2016 [6]	Examined systemic inflammatory markers like IL-6 and TNF-α during CPB	IL-6 and TNF-α elevations contribute significantly to atrial remodeling and arrhythmogenesis
Arslan et al., 2021 [11]	Studied inflammatory markers (NLR, PIV, etc.) correlating with adverse outcomes	Higher NLR and PIV levels are associated with prolonged ventilation and ICU stay
Chacon-Alberty et al., 2023 [12]	Investigated IL-6, TNF-α, and vascular injuries during CPB in lung transplantation	Vascular injuries and immune activation contribute to systemic inflammation during CPB

**Table 2:** Summary of key studies examining cardiopulmonary bypass (CPB) and aortic cross-clamping (ACC) effects on postoperative atrial fibrillation (POAF). CABG: coronary artery bypass graft; ICU: intensive care unit; IABP: intra-aortic balloon pump; EcMO: extracorporeal membrane oxygenation; AKI: acute kidney injury; RRT: renal replacement therapy; PCI: percutaneous coronary intervention; AF: atrial fibrillation; MACEs: major adverse cardiac events; N/A: not available.

Study	Type of Study and Patient Population	Aim	Outcomes	Focus	Key Mechanism	Definition, Duration, and Type of POAF	Perioperative Use of Antiarrhythmic Drugs
Mohamed et al., 2017 [37]	A prospective, non-randomized, single-center study which included 30 patients undergoing isolated on-pump CABG surgery	Investigation of the ACC’s effect on the mechanical ventilation postoperatively	Duration of mechanical ventilation, postoperative ischemia, postoperative myocardial infarction, re-exploration, neurologic dysfunction, renal impairment, chest infection and respiratory failure, length of ICU stay, in hospital length of stay, early mortality	ACC >60 min linked to systemic complications like renal dysfunction; POAF is not mentioned as an outcome	Systemic inflammation	N/A	N/A
Ruggieri et al., 2018 [36]	A prospective, multicenter study which included 2962 patients undergoing isolated CABG operation	Investigation of the prognostic impact of prolonged ACC time	In-hospital/30-day mortality, prolonged inotropic support, postoperative use of IABP and EcMO, sternal wound infection, AKI, RRT, delirium, stroke, resternotomy for bleeding, need for PCI, atrial fibrillation, ICU stay, and the composite outcome E-CABG complication grades 3–4 *	Prolonged ACC (>75 min) associated with increased POAF and 30-day mortality	Ischemia–reperfusion injury	N/A	N/A
Erkut and Ates, 2019 [38]	A prospective, single-center study which included 91 patients undergoing isolated and elective CABG surgery	Evaluation of the changes for troponin I levels during surgery and determination of the correlation between those and the ACC time	Need for inotropic support, AF, pulmonary dysfunction failure without intubation, defibrillation for ventricular fibrillation, postoperative IABP application, re-intubation, diuretic need, neurologic dysfunction, death, infections associated with sternotomy and mediastinal region, gastrointestinal dysfunction, and renal dysfunction	ACC > 50 min associated with elevated troponin I and myocardial injury	Myocardial injury (troponin I as marker)	N/A	N/A
Swinkels et al., 2021 [39]	A retrospective cohort study, which included 456 consecutive patients who had undergone isolated aortic valve replacement	Identification of possible independent predictors of decreased late survival, including ACC and CPB	In-hospital MACEs **, late MACEs **, and late survival (until 30 years after surgery)	ACC predicts reduced long-term survival due to cardiac mortality	Long-term cardiac remodeling and mortality	New-onset or new, paroxysmal or permanent	N/A
Dayi et al., 2023 [40]	A single-center prospective study which included 103 consecutive patients who underwent isolated CABG	Identification of the effect of ACC time on the development of POAF	Incidence of POAF	No significant association between ACC and POAF	No direct ACC–POAF link	Any AF episode that lasted > 15 min at ECG monitoring or needed therapy for hemodynamic instability was defined as POAF.	All the cases used b-blockers at least 24 h before the operation.
Khassawneh et al., 2023 [41]	A single-center, retrospective study, which included 100 low-risk patients for arrythmia *** undergoing elective cardiac surgery	Evaluation of the correlation between prolonged ACC time and new onset of cardiac arrhythmia (atrial tachyarrhythmias, bradyarrhythmias, and ventricular arrhythmias)	Incidence of new-onset of cardiac arrhythmia	ACC > 90 min minimally impacts arrhythmias	Limited myocardial stress in low-risk cases	New-onset of cardiac arrhythmia (includes AF episodes) within the first 48 h postoperatively	N/A

* The E-CABG complication grades 3–4 was employed as a composite measure of adverse outcome and includes transfusion of >10 units of red blood cells, renal failure requiring dialysis, mediastinitis, stroke, early repeat revascularization, reoperation for hemodynamic instability, ventricular fibrillation/asystole, surgery for gastrointestinal complications, the need of extracorporeal membrane oxygenation, and/or in-hospital/30-day death. ** In-hospital MACEs: new-onset AF, systolic heart failure, inotropic therapy, adult respiratory distress syndrome, dialysis, sepsis, mediastinitis, cardiac tamponade, ischemic stroke, non-fatal myocardial infarction, re-thoracotomy, permanent pacemaker implantation, and endocarditis. Late MACEs: atrial fibrillation, systolic heart failure, ischemic stroke, hemorrhagic stroke, non-fatal myocardial infarction, permanent pacemaker implantation, and endocarditis. *** Patients with no previous record of coronary artery disease, connective tissue disease, scarring from previous heart attack (or heart failure), high blood pressure, a normal or near-normal electrocardiogram, and negative initial cardiac injury markers.

## Data Availability

Not applicable.

## Abbreviations

The following abbreviations are used in this manuscript:

POAF	Postoperative atrial fibrillation
CABG	Coronary artery bypass grafting
CPB	Cardiopulmonary bypass
ACC	Aortic cross-clamping
CC	Cross-clamping
IL-6	Interleukin 6
TNF-α	Tumor necrosis factor alpha
NLR	Neutrophil-to-lymphocyte ratio
PIV	Pan-immune inflammatory value
ICU	Intensive care unit
DNC	Del Nido cardioplegia
CK-MB	Creatine kinase MB
ROS	Reactive oxygen species
AVR	Aortic valve replacement
AF	Atrial fibrillation
IABP	Intra-aortic balloon pump
EcMO	Extracorporeal membrane oxygenation
AKI	Acute kidney injury
RRT	Renal replacement therapy
PCI	Percutaneous coronary intervention
MACE	Major adverse cardiac events
NWR	Neutrophil-to-white cell ratio
Hs-TnT	High-sensitivity troponin T
CRP	C-reactive protein
miRNA	MicroRNA
CADM1/STAT3	Cell adhesion molecule 1/transducer and activator of transcription 3
SERPINH1	Serpin Family H Member 1
BNP	B-type natriuretic peptide
PPARα	Peroxisome proliferator-activated receptor alpha
